# THE INFLUENCE OF SOCIODEMOGRAPHIC FACTORS ON GENDER DISPARITIES IN DISABILITY TRENDS

**DOI:** 10.1093/geroni/igz038.926

**Published:** 2019-11-08

**Authors:** Ya-Mei Chen, Hsiao-Wei Yu, Tung-Liang Chiang, Duan-Rung Chen, Yu-Kang Tu

**Affiliations:** 1 National Taiwan University, Taipei, Taiwan; 2 Chang Gung University of Science and Technology, Taipei, Taiwan

## Abstract

Objective. In Taiwan as well as in many other aging societies, decreasing disability is a key public health priority. Gender is known to be a significant factor for developing disability. Our study aimed to examine gender disparities in disability trends as well as how sociodemographic factors influence these disparities. Methods. We used multiple-group latent growth curve modeling (MG-LGM) to examine data drawn from the Taiwanese Longitudinal Study of Aging, a longitudinal and nationally representative survey database. Four waves of survey data and 3,429 older adults (mean ages = 50-96) were included for analysis. Disability trajectories among men and women were modeled separately using MG-LGM. Equality constraints were imposed on the six factors assessed: age, education, leisure activities, perceived (self-rated) health, health behaviors, and comorbidities. Results. Baseline disability levels were not significantly different between the two groups, but once disability began, the progression toward greater disability was almost 50% faster among older women. Greater age and more comorbidities added significantly more to baseline disability and speed of progression among older women than among older men (p < 0.001). However, having better health behaviors (e.g., no alcohol, more leisure activities) reduced baseline disability significantly more among women (p < 0.05). Particularly interesting findings include that perceived health reduced baseline disability only among men (p < 0.05), while having a better social network reduced baseline disability only among women (p < 0.05). Conclusion. For older women, disability prevention is crucial, and promoting positive health behaviors and strong social networks are promising strategies.

